# Bigels as Delivery Systems: Potential Uses and Applicability in Food

**DOI:** 10.3390/gels9080648

**Published:** 2023-08-11

**Authors:** Alyssa Francavilla, Maria G. Corradini, Iris J. Joye

**Affiliations:** 1 Department of Food Science, Ontario Agricultural College, University of Guelph, Guelph, ON N1G 2W1, Canada; afrancav@uoguelph.ca (A.F.); mcorradi@uoguelph.ca (M.G.C.); 2 Arrell Food Institute, University of Guelph, Guelph, ON N1G 2W1, Canada

**Keywords:** bigels, hydrogels, organogels, controlled release, solid fat replacement, food

## Abstract

Bigels have been mainly applied in the pharmaceutical sector for the controlled release of drugs or therapeutics. However, these systems, with their intricate structures, hold great promise for wider application in food products. Besides their classical role as carrier and target delivery vehicles for molecules of interest, bigels may also be valuable tools for building complex food structures. In the context of reducing or even eliminating undesirable (but often highly functional) food components, current strategies often critically affect food structure and palatability. The production of solid fat systems that are *trans*-fat-free and have high levels of unsaturated fatty acids is one of the challenges the food industry currently faces. According to recent studies, bigels can be successfully used as ingredients for total or partial solid fat replacement in complex food matrices. This review aims to critically assess current research on bigels in food and pharmaceutical applications, discuss the role of bigel composition and production parameters on the characteristics of bigels and further expand the use of bigels as solid fat replacers and functional food ingredients. The hydrogel:oleogel ratio, selected gelators, inclusion of surfactants and encapsulation of molecules of interest, and process parameters (e.g., temperature, shear rate) during bigel production play a crucial role in the bigel’s rheological and textural properties, microstructure, release characteristics, biocompatibility, and stability. Besides exploring the role of these parameters in bigel production, future research directions for bigels in a food context are explored.

## 1. Introduction

Consumer demand for convenient and shelf-stable food products with good nutritional quality and specific health benefits has increased in recent years. However, the development of “health-improving” food products is not always straightforward due to the necessity of striking a balance among improved nutritional and nutraceutical profiles, consumer expectations for convenience and sensory attributes, stability, and regulatory requirements. In addition to food fortification with bioactive components, the avoidance or reduction of so-called “bad nutrients” (although often highly functional) such as sugar, sodium, and saturated/*trans*-fat is a rising trend [1]. Additionally, the World Health Organization’s goal of globally eliminating artificially produced *trans*-fatty acids from foods by the end of 2023 has put this topic even higher on the sector’s innovation agenda [2].

Fortifying food with bioactive components is not a simple endeavor. Bioactive components are often labile and/or might have adverse effects on the organoleptic properties of food products. Hence, colloidal delivery systems have been customarily developed to encapsulate, protect, and release labile compounds in pharmaceutical, food, and cosmetic applications [3]. In foods, these compounds usually include vitamins, antimicrobial agents, pre/probiotics, antioxidants, flavors, and colors [3,4,5]. The inclusion of these compounds in delivery systems ensures that they are fully dispersed in the food matrix, protected against degradation, and/or sufficiently released/absorbed in the desired location (e.g., the gastrointestinal tract) to enhance their bioaccessibility and bioavailability while minimally affecting food quality [6,7]. While numerous delivery systems have been explored, gel-based and specifically biphasic gelled delivery systems are promising structures that can be used to protect and release labile bioactive molecules in a complex matrix. Gels are semi-solid structures with high amounts of liquid entrapped in a three-dimensional network [6]. Gels can be categorized by the polarity of the liquid phase, with hydrogels having a polar liquid phase (water) and organogels having an apolar liquid phase (e.g., oil). Organogels composed of edible ingredients are termed “oleogels”. These terms, i.e., organogel and oleogel, will be used interchangeably where appropriate. The combination of a hydrogel and organogel gives rise to a biphasic gelled structure, often referred to as a bigel. Bigels are promising delivery vehicles for bioactive molecules in food due to several key characteristics:

i. Both phases are gelled, and thus bigels have high physical stability, and their rigidity and elasticity can provide texture and structure to foods [8];
ii. Their biphasic nature allows for the (co-)delivery of a wide range of components;
iii. Their physicochemical and release properties can be fine-tuned by changing the composition and structure of each phase [9].

**Figure 1:**
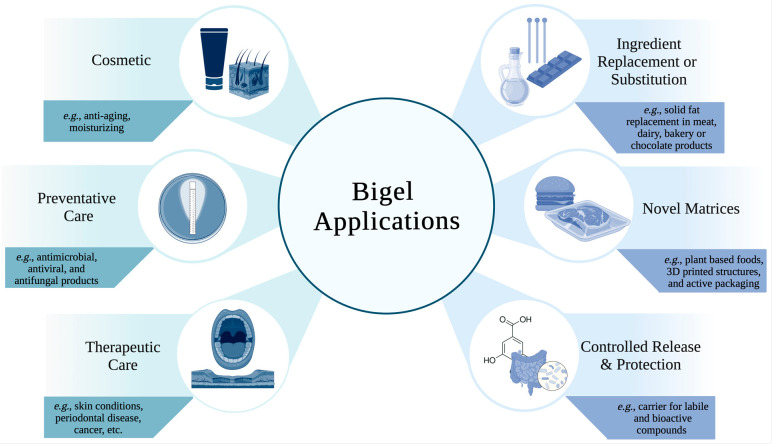
Current pharmaceutical (**left**) and food (**right**) applications of bigels. Illustration created with biorender.com.

Bigels have been widely studied as drug delivery vehicles, particularly for transdermal applications. In food products, bigel applications have been very limited and largely restricted to solid fat replacers and delivery vehicles for functional compounds. Hence, based on the unique properties and potential of bigels, more research is needed to expand their food uses. Figure 1 illustrates the current applications of bigels in food and pharmaceuticals. This review will, therefore, critically summarize the current research on bigels in foods and pharmaceuticals, and explore potential future directions and research areas for bigels in foods.

## 2. Bigels: Definition, Preparation Methods, and Characterization

Bigels, also referred to as biphasic or hybrid gels, are an emerging class of soft materials composed of two discrete gel phases, typically a hydrogel and an organogel, where both gel phases contribute to the physical properties of the material [10]. Physicochemical characteristics, rheological properties and delivery capabilities of individual hydrogels [11,12,13] and organogels [14,15,16,17,18] have been extensively reported [19]. The insights gained from this research on individual gels will aid in designing bigels with specific properties. Despite being combined, the resulting bigel structures retain some of the characteristics of the individual systems, while also often resulting in functional enhancements over the individual parts [10,20]. Bigels benefit from having both hydrophilic and lipophilic phases, making them suitable to encapsulate and deliver both hydrophilic and lipophilic active agents. In topical applications, organogels have been reported to hydrate the stratum corneum, improving the penetration of drugs into the skin [21]. Additionally, bigels are often easy to prepare, spread and wash [10,20,21]. By combining two gel phases with different structures and at different proportions, both the physicochemical and release properties can be tuned. The system’s physical stability can also be improved due to the entrapment of both liquid phases within a network (in comparison to an emulgel, which has only one structured phase) [15,22,23]. Due to the vast tunability of the systems and the parameters that can be modulated throughout the production process, bigel properties are currently difficult to predict. Hence, in-depth studies on structure–function relationships are needed [22].

**Figure 2:**
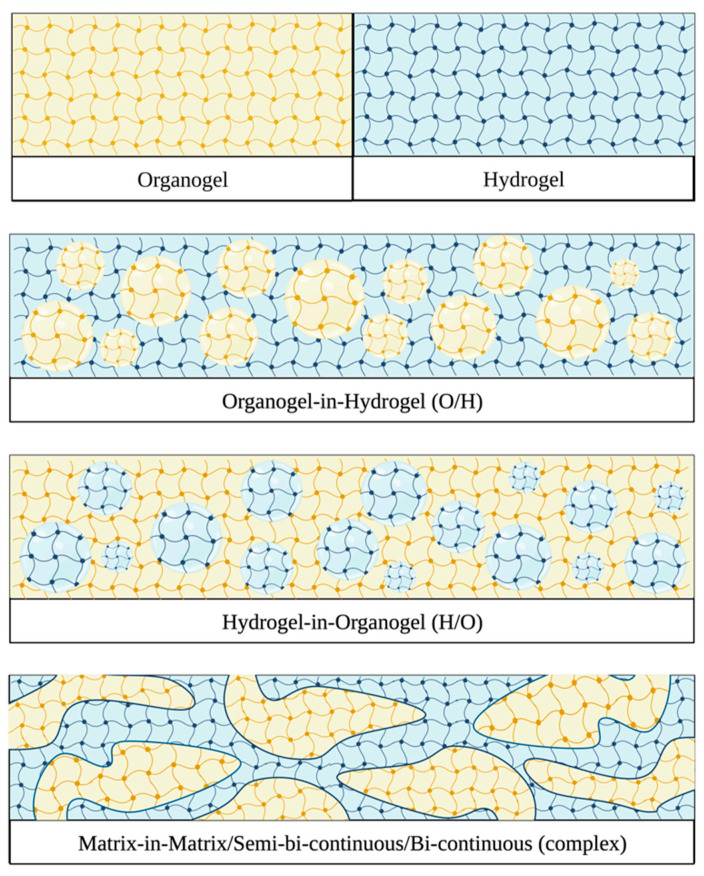
Schematic illustration of organogels, hydrogels, and bigel matrices. Illustration created with biorender.com.

Depending on the distribution of the individual gel phases (organogel and hydrogel) within the bigel, these systems can be classified as (1) organogel-in-hydrogel (O/H), (2) hydrogel-in-organogel (H/O), or (3) complex (bicontinuous/semibicontinuous/matrix-in-matrix) structures (Figure 2) [10,20]. O/H bigels have thus far been the most extensively studied. Recently, H/O and complex bigels have also received more attention [24,25]. Besides their specific composition, the phase distributions of the components can also contribute to the physicochemical and release properties of bigels, which will be explored in the following sections.

### 2.1. Composition and Production Methods

**Figure 3:**
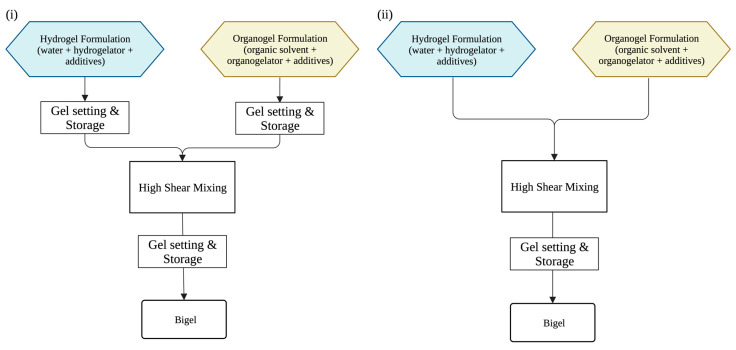
Flow diagram for bigel production via two main mixing methods. (**i**) Individual hydrogels/organogels are set, and then combined by high shear mixing. (**ii**) The bigel is produced via high shear mixing of the hydrogel and organogel. Illustration created with biorender.com.

Bigels have been produced via two main methods, where (i) the molten organogel and hydrogel are combined at high shear followed by gel setting, or (ii) both gels are prepared and allowed to set prior to high-shear mixing (Figure 3) [10]. The preparation of the individual gels is generally a simple process wherein the hydrogelator/organogelator is dispersed/dissolved into the liquid phase. Then, the gelling mechanism is triggered based on the gelator’s requirements.

Gelators can be classified based on their molecular weight as either low or high molecular weight or polymeric gelators [16,18]. Low-molecular-weight (<1 kDa) gelators (LMWG) are able to self-assemble through noncovalent bonds at concentrations as low as 2%. These physical interactions lead to large aggregates that interweave and cause the gelation of the matrix through chemical crosslinking [16,18]. These networks can either be strong or weak [16]. High-molecular weight (>2 kDa) gelators, or polymeric gelators, typically gel at lower concentrations (<2%) than LMWGs. Gels of high molecular weight organogelators can be both physical (non-covalent interactions) or chemical (crosslinked) gels [16,18]. Most LMWG identified to date perform better in non-polar solvents [26]. Therefore, the most commonly used hydrogelators are polymeric, although hydrophilic LMWGs have been identified [27]. In drug delivery, gelators only require biocompatibility, but for food applications, they must be edible and approved for food use by the relevant regulatory agencies. In both hydro- and organogels, gelator selection is important to obtain the proper desired qualities of the gel.

Chemical organogels are less sensitive to temperature due to the formation of a supramolecular polymer network through non-covalent bonds [18]. However, organogels (especially physical organogels) typically undergo solid-to-liquid transitions when the temperature is above their sol-to-gel transition temperature [16]. According to the literature, numerous organogelators have been used to formulate bigels, including sorbitan esters (e.g., sorbitan monostearate and sorbitan monopalmitate), monoglycerides and fatty acids, waxes, lecithin, and others [10]. The liquid phases used are also subject to regulatory approval. While the liquid phase options for pharmaceutical applications are relatively broad, the organic liquid phases acceptable for food bigel formulation are largely restricted to vegetable and seed oils (e.g., canola, sesame, olive oil). Typically, organogelation occurs by heating the liquid phase/organogelator mixture to a required temperature, prior to cooling the mixture to obtain the gel.

Polymers for hydrogelation can be natural, semi-synthetic, or synthetic. Regardless of their origin, all should be able to form a polymer network that can bind large quantities of water [8]. Natural polymers such as chitosan, gellan, pectin, xanthan gum, guar gum, starch, locust bean gum, alginate, agarose, collagen, and gelatin have previously been used for bigel production [8,10]. Synthetic polymers like carbomers, poloxamer, polyvinyl alcohol, polyethylene oxide, and polyacrylic acid have also been used to produce tunable hydrogels. Still, these polymers do not have applications in food-grade bigels [10]. Finally, semi-synthetic polymers such as methylcellulose and hydroxypropyl methylcellulose (HPMC), have also been utilized in bigel preparation [10]. Hydrogelation can occur through physical interactions (transient intersections caused by physical entanglements and weak forces, e.g., hydrogen bonds) and/or chemical cross-linking. These polymers form gels with differing physical characteristics and are responsive to physical triggers (temperature, pH, enzymes, etc.) for release.

Surfactants can also be included in the formulation and have been shown to affect the rheological properties of the bigel. For example, sucrose esters with differing hydrophilic–lipophilic balance (HLB) values were incorporated into an O/H bigel to modulate its rheological properties [28]. The addition of surfactants with lower HLB values resulted in samples with higher solid-like and elastic behavior. This was attributed to the overall smaller size of the dispersed organogel particles in the systems [28]. Not only the HLB values but also the type and concentration of surfactants are important. In O/H bigels where mono-diglycerides were added, increasing the surfactant concentrations resulted in a phase inversion to a H/O bigel [29]. Additionally, the mono- and diglycerides negatively affected the hardness of the bigel in contrast to the addition of sucrose esters [29].

The inclusion of active agents can also impact gel properties. Polyphenols, for example, occasionally cause biopolymer aggregation, which may lead to an increase or decrease in the network strength during hydrogel formation [30]. In bigels containing aqueous *Quercus resinosa* polyphenol extracts, an increase in polyphenol concentration increased the consistency index of the bigel [30]. Similarly, the addition of vitamin E had a sigmoidal, dose-dependent positive impact on the gel strength and phase transition temperature of a 12-hydroxystearic acid/candelilla wax organogel [31].

Preparation conditions must be tightly controlled during bigel production. The preparation temperature, for example, is important when incorporating thermo-labile active agents into the gels. Yet, temperature may also impact the mechanical and rheological properties and structural characteristics of the final bigel [32]. The gels can be mixed together while still molten (>70 °C, Figure 3), which usually results in a more homogenous distribution of phases [32,33]. The homogeneity could be attributed to the liquid form of both phases at high temperatures. Alternatively, both gels can be formed separately and then mixed together after gelling and storage (Figure 3) [21]. The second method may result in a more complete gelling of the individual phases, but could also result in less overall stability and homogeneity. However, if the formulation includes sensitive active agents, or thermally unstable hydrogelators, the second method is deemed more suitable due to the shorter exposure of the active agent to harsh conditions [23].

The shear rate during mixing also affects the particle size of the dispersed phase (gel discrete particles within the continuous gel matrix) [34]. In H/O bigels with a constant hydrogel volume, a small (~40 µm) particle size of the hydrogel pointed to an active filler effect in the continuous oleogel phase [29]. Small hydrogel particle sizes resulted in final H/O bigels with the strongest rheological and textural properties when compared to medium and large particle-sized hydrogel bigels [34]. Additionally, small particle sizes enhanced the oil binding capacity of the oleogel. Tailoring the dispersed phase’s particle size is therefore necessary for the thorough design of a bigel with ideal properties for the desired application.

The hydrogel–oleogel ratio can impact the type (i.e., O/H, H/O, or complex), textural, rheological and structural properties of the bigel [15]. In general, bigels with increasing O:H ratios are characterized by a decreasing firmness and spreadability and an increasing adhesiveness and cohesiveness [35]. It was also observed that the organogel rheological properties dominated the properties of beeswax/alginate O/H bigels [35]. In another H/O bigel system, an increase in organogel content resulted in more crystalline systems with enhanced thermal stability, a softer texture, lower viscosity, and good plasticity/spreadability [36]. Conversely, increased hydrogel content resulted in higher viscosity and bigel strength [36].

Finally, storage time also influenced bigel properties [37]. In bigels prepared with monoacylglycerides as the oleogelator, refrigerated storage for 15 days improved the mechanical properties of the bigel due to the slow structuring of monoacylglcerides [37]. Prior to the 15-day point, the bigel firmness had not reached the maximum potential value, indicating the potential for storage time as a parameter that should be controlled in bigel manufacture [37].

### 2.2. Characterization

**Figure 4:**
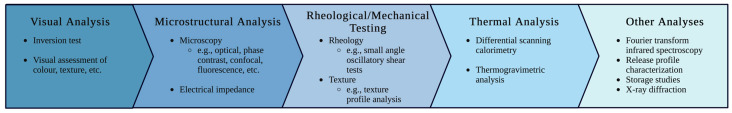
Schematic overview of analytical techniques commonly applied during bigel characterization. Illustration created with biorender.com.

Analytical methods can be applied to properly characterize the produced bigel, and to ascertain its functionality. Several techniques are commonly used to assess bigel properties, but the initial step performed is an inversion test, to determine the formation of a “correct” bigel (that can stand under its own weight, or behave as a solid under gravitational pull). The most common analytical techniques are further described in the following sections (Figure 4).

#### 2.2.1. Microstructural Analysis

Microstructural analyses are frequently carried out to study the morphology of bigels. Microscopy is a simple characterization technique encompassing several modes with distinct suitability for analyzing different bigel types. Confocal laser scanning [24,32,34,38,39,40,41], phase contrast [23], optical [24,30,38,42] (including fluorescence [35,43] and polarized light [34,36,37,42,44]), transmission electron [45], and scanning electron [46,47] microscopy have been used for this purpose. All microscopy techniques provide insights into the microstructure of the bigel and, more specifically, the arrangement of the phases. However, unique microscopy techniques could be selected to gain additional information about the materials. For example, micro-spectroscopy techniques such as Raman microscopy could provide spatial chemical mapping of the material without staining the samples, potentially lending insights into the distribution of active agents in the bigel [48]. Microscopes with heated stages could allow for the visualization of the melting behavior of bigels for saturated fat-replacer applications and controlled release strategies relying on thermal triggers.

#### 2.2.2. Rheological and Mechanical Testing

The rheological and mechanical properties of bigels are commonly used to evaluate the quality and utility of the produced bigels, since these parameters directly impact the commercial applicability of the products.

The flow behavior and viscosity of the gels are significantly affected by the organo/hydrogelator molecular weight, concentration, and structure (e.g., branched vs. linear polysaccharide gelators) [22]. Small-Amplitude Oscillatory Shear (SAOS) tests are typically used to study the viscoelastic properties of gels under small deformations. They allow us to gain insights into the (type, strength and number of) interactions and molecular/aggregate/microstructures underlying the overall gel structure [22]. Prior to SAOS testing, a strain sweep test would be utilized to determine the linear viscoelastic region, which allows for the identification of the extent of deformation the gel structure can experience before structural breakdown or yielding becomes apparent. A stress sweep test would allow for the determination of the yield stress associated with the gel structure. Larger deformations may be applied with a creep test. In this test, the response of the gel to a constant stress provides information on the viscoelastic behavior of the gel under moderate deformations. After stress removal, the percent recovery of the gel is related to the elastic behavior or the storage of the deformation energy in the gel structure. As with SAOS measurements, these tests give indirect information about the interactions and molecular conformations underlying the overall gel structure.

The bigel textural properties, such as firmness, cohesiveness, adhesiveness, and spreadability, have been derived from compression–decompression tests like texture profile analysis (TPA) [10,20,22]. These parameters are important in both pharmaceutical and food applications. In drug delivery applications, e.g., a topical cream, the texture of the gel is central to patient use and compliance. In food applications, e.g., solid fat replacers, the bigel must replicate the rheological and textural properties of the replaced fat. Pastry shortening, for example, is a key ingredient in puff pastries with a significant role in the formation of the final product’s structure through the lamination process [32]. The bigel, therefore, needs to be able to form a continuous layer on the dough, withstand high shear rates and extensional deformation, and mimic the flow behavior, firmness, and melting profile of the replaced solid fat [32]. Therefore, rheological and textural testing are of great importance in screening potential bigel formulations for fat replacers and other uses.

Although the organogel and hydrogel properties impact the final rheological properties, bigels are ultimately multiphasic materials whose overall rheological behaviour depends on many factors, such as each separate phase properties, the particle size distribution and the volumetric fraction of the dispersed phase [22]. The exact relationship and interplay between any or all these factors and bigel rheological behavior have not been fully elucidated yet. However, empirical modifications to established theoretical rheological models for complex gelled systems have been developed [49,50]. They can eventually be used to better understand the contributions of hydrogels and organogels to bigel rheology [51].

#### 2.2.3. Other Characterization Methods

In addition to the microstructural, rheological and textural properties, other characteristics dictate the usability of bigel formulations for specific applications. Thermal analysis, for example, allows for determining the thermal stability/melting temperature of the bigel formulation. The temperature-dependent transformation from solid-like to liquid-like behavior is critical for practical applications in both the pharmaceutical and food realms. This transformation impacts the release profile of enclosed components, and the structural characteristics of the bigel (e.g., baking pastry with a saturated fat replacer). A temperature sweep test utilizing a rheometer, as described above, will allow the “visualization” of the phase transitions as a function of temperature through studying the viscoelastic parameters [52]. The actual phase transition between solid and liquid structures can also be studied by differential scanning calorimetry. For this test, a weighed amount of the bigel is heated or cooled over a temperature range, and the heat flow is recorded for the bigel and compared to the heat flow to a reference material undergoing the same heating or cooling profile. Endo- and exothermic peaks indicate melting or crystallization processes. Specifically, the temperatures at which these transitions occur will be of interest to the applicability of the bigel [33]. Thermogravimetric analyses can also be performed, providing information about thermal events such as melting and water evaporation [53].

Fourier transform infrared (FTIR) spectroscopy has been applied to identify functional groups on polymers, and chemical interactions between bigel components themselves and with enclosed molecules of interest. For example, in a Carbopol^®^ 934/sorbitan monostearate and sesame oil bigel, the characteristic peaks of the raw materials closely reseembled those of the separate systems, indicating the absence of chemical interactions between the two polymers [43]. X-ray diffraction is another useful tool used to elucidate whether the bigel structures are amorphous or contain crystalline substructures [54]. Electrical impedance has also been used to assess changes in microstructure caused by, for example, different O:H ratios of the material based on the conductivity of the phases and their interconnectivity [44]. Impedance values depend on the diffusion of solutes within the gel matrices. Higher impedance is associated with low diffusion coefficients for solutes, provided that interconnected channels are present [44].

Analysis can also be performed to determine the release characteristics and related properties of the bigel. In pharmaceutical applications, this testing is typically performed under both in vivo and in vitro conditions. Mucoadhesion [55,56,57], in vivo and ex vivo skin permeation [53,58,59], in vitro cytocompatibility [57,59], and in vivo drug release [43,45] are all useful tests to assess the behavior and efficacy of bigels as delivery systems. Modified Franz cells are common apparatuses used to measure drug release, penetration and diffusion. They can measure mucoadhesion, depending on the barrier material used [10,43]. The bioaccessibility and bioavailability of active agents in the gastrointestinal tract can also be measured using in vitro digestion models [60]. Drug release via diffusion can be effectively estimated based on Fick’s law [10]. The swelling of the gel structure will widen the channels and pores in a gel and aid in diffusion [33]. The hydrogel portion of the bigel dictates the swelling behavior. Factors affecting the swelling include temperature, pH, and ionic strength. Moreover, the presence, proportion, and distribution of organogels in the bigel tend to control the swelling and thus the release of the encapsulated molecules, especially due to the lack of swelling of the organogel structure [10,55].

Although bigels exhibit greater physical stability than similar colloids, biphasic systems can still undergo destabilization over time. Therefore, their stability should be assessed. Storage studies are used to evaluate changes in microstructural, thermal, and rheological properties over a predetermined time at a preset temperature profile [10]. Accelerated photostability tests have also been performed, where the quantity of a photo-labile active agent (e.g., ketoprofen) was measured after exposure to daylight [45]. The amount of the encapsulated compound remaining in the bigels after storage under selected scenarios has seldom been reported, but it is important for establishing bigels as effective materials for diverse applications.

## 3. Current Pharmaceutical Applications

Bigels for pharmaceutical use have been extensively studied. Drug carriers allow for obtaining the required therapeutic effects by protecting the enclosed compounds until they are released from the bigel into the targeted site. Transdermal/topical delivery is one area where bigels have been investigated due to the numerous benefits of bigels in terms of spreadability, cooling and moisturizing effects, water washability from the skin, and stability during storage [10,20]. Skin is the largest organ of the body, and topical delivery is advantageous because it is non-invasive, does not require training, and presents a lower health risk compared to other delivery strategies currently used for pharmaceutical purposes (e.g., intravenous delivery) [20]. To attain appropriate topical delivery using bigels, it is crucial to understand their flow behavior, drug permeability through the skin, and the physicochemical properties of the entrapped drugs [20]. In addition to topical delivery, nasal, rectal, buccal, oral, and parenteral delivery applications are promising uses for bigels [10]. Different triggers (e.g., pH, temperature, and enzymes) for drug release can be exploited, depending on the treatment requirements and the gelling mechanisms of the bigel. However, to this day, most bigel drug delivery applications have relied on diffusion-mediated release only. Table 1 summarizes different bigel systems with pharmaceutical applications.

Foundational studies on the role of bigel formulation on pharmaceutical drug release profiles have been performed. Tamarind gum hydrogel/stearic acid and rice bran oil oleogel bigels at different O:H ratios resulted in bigels with various microstructures and eventually phase inversion (O/H vs. H/O) [41]. These diverse microstructures (O/H, H/O, or complex) were detected based on changes in the electrical impedance of the formulations. The results suggest a change in diffusion properties of the studied solute (moxifloxacin HCl was used as the model drug), therefore impacting drug release [41]. In another formulation, ciprofloxacin (an antimicrobial) was incorporated into a gelatin hydrogel/Span 60 and sesame oil oleogel bigel [33]. Drug release was also diffusion-mediated in this case, and swelling was impeded in the bigel compared to a separate hydrogel [33]. The inclusion of date palm-derived cellulose nano-crystals in a guar gum hydrogel/candelilla wax and sesame oil oleogel bigel (loaded with moxifloxacin HCl) altered the physical and biochemical properties of the bigel [61]. Increasing nano-crystal concentration increased the firmness of the bigel, and changed the drug release profile in a non-dose dependent manner, indicating that the inclusion of nano-crystals or other fillers could aid in fine-tuning the delivery systems [59]. Sodium alginate or HPMC hydrogels/beeswax and fish oil bigels were prepared to assess the permeation of imiquimod (used in skin disease treatment) across the skin barrier [53]. The bigels were effective transdermal delivery vehicles, and the presence of an oil phase improved skin permeation [53]. The bigel structure also effectively protected the polyunsaturated fatty acids (particularly EPA and DHA) in fish oil from oxidation [53].

As previously mentioned, the type and properties of the starting materials also modulate the final properties of the bigel. Bigels containing different combinations and concentrations of hypromellose, collagen, gelatin, alginate, sesame oil, medium-chain triglycerides, and isopropyl myristate produced through different mixing regimes displayed distinct release profiles and physicochemical properties. These observations emphasize the critical effect of starting materials and processing conditions on the bigel properties [69]. The effects of emulsifiers [42,70] and additives (e.g., antioxidants) were also evaluated [71]. For example, the incorporation of polysorbate 80 in a poloxamer hydrogel/mineral oil and fumed silica oleogel bigel led to a decrease in the sol–gel temperature, slower drug (ciclopirox olamine) release, and better freeze–thaw stability [70]. Similarly, the addition of vitamin E increased the diameter of dispersed phase droplets. However, vitamin E had no effect on the rheology, microstructure, biological behavior, and oxidative stability of bigel formulations consisting of various mixtures of candelilla wax, 12-hydroxystearic acid, mineral oil, and/or sunflower oil with acrylate polymer hydrogels [42]. Novel thermal treatments have been the subject of publications on bigel processing conditions. The use of microwave heating to prepare a thymoquinone-loaded Carbopol^®^ hydrogel/PEG 400 oleogel bigel did not negatively impact the physicochemical or release properties of the bigel. Microwave heating was more energy-efficient than conventional heating [71].

### 3.1. Cosmetics and Topical Treatments

Cosmetic applications for bigels have been widely reported, their moisturizing effect being of primary interest. Several bigel formulations were prepared from different oleogels (Span 60 and sweet almond oil, cholesterol and liquid paraffin, zinc stearate and paraffin, and silicic acid and sweet almond oil) mixed with a hydrogel (Carbopol^®^ and triethanolamine) [21]. The bigels’ moisturizing effect was measured hourly using a corneometeror five hours after application [21]. Carbopol^®^ hydrogel/sorbitan monostearate and almond oil oleogel bigels were formulated at different ratios, and were also found to be innocuous and moisturizing [72]. The moisturizing effect of all tested formulations points to their excellent performance as topical cosmetic formulations [21]. Current bigel skincare formulations can also be modified to provide a better delivery system for cosmetic agents (e.g., anti-aging compounds). Enriching a previously established cosmetic formulation with monoglycerides modified the microstructure of the bigel, potentially impacting the delivery properties of the bigel [52]. Coenzyme Q10 (CoQ10) is a popular skincare ingredient. It is a vitamin-like oil-soluble molecule with antioxidant and anti-aging effects [58]. Building on previous research [53], CoQ10 was incorporated into both the aqueous and lipid phases of a Carbopol^®^ hydrogel/beeswax and fish oil oleogel bigel [58]. The optimal bigel formulation effectively delivered CoQ10 and fish oil fatty acids through the skin [58]. The combination of viscoelastic and lipophilic properties of the bigel were important for skin permeation [58].

In addition to topical cosmetic applications, topical treatments for skin conditions have been investigated using bigels. Acne is an extremely prevalent skin disease worldwide and requires the development of an effective topical treatment that encourages patient compliance [64]. Doxycycline hyclate has been investigated as an acne treatment using a bigel (Carbopol^®^ 940 hydrogel/Span 60 and olive oil oleogel) with ~70–80% encapsulation ability [64]. In vivo rabbit ear models treated with the developed bigel showed a bigger decrease in acne dimensions compared to a commercially available acne treatment [64]. Antifungal formulations are also suitable for transdermal delivery. Novel bigel formulations as a carrier for *Bidens tripartita* essential oil (an antifungal agent) were tested for antifungal efficacy, bioadhesion, and skin toxicity [62]. These bigels were found to be promising delivery systems for antifungal agents [62]. Ciclopirox olamine and terbinafine hydrochloride are two other antifungal compounds [63]. Bigels prepared from poloxamer 407 hydrogel/polyethylene and liquid paraffin organogel were good carriers of these two drugs [63]. The release of the drugs could be modified by varying the oleogel concentration and the phase in which the drugs were incorporated [63]. Even with lower total drug release, all bigel formulations significantly inhibited *Microsporum canis* [63]. Skin cancer can also be treated topically, and bigels have been investigated for this application as well. Building on previous fish oil-based bigel research, the incorporation of imiquimod into a bigel (Carbopol^®^ hydrogel/beeswax and fish oil oleogel) was investigated as a treatment for skin cancer [59]. Imiquimod is a potent skin cancer treatment, also associated with severe skin inflammation. Fish oil was added to inhibit this undesirable side effect [59]. The bigel formulation was more effective than other tested delivery vehicles (separate hydrogel and oleogel formulations) because it exhibited higher drug availability in the skin, anti-tumor effects, and reduced pro-inflammatory cytokine levels [59].

Hand hygiene is an area of increasing research interest, especially in recent years. While there are tools to improve hand hygiene and prevent the spread of infectious microorganisms, continued disinfection using ethanol-based hand disinfectants can cause hand irritation and result in decreased compliance [65]. Povidone-iodine is an extremely effective hand sanitizer, but it requires frequent re-application [65]. Therefore, a bigel (HPMC hydrogel/beeswax and alpha-tocopherol and olive oil oleogel) loaded with povidone-iodine was prepared, which sustained antiseptic action for an hour after application [65]. The moisturizing effect of bigels could increase the compliance of hand disinfection practices compared to traditional hygiene products [65].

### 3.2. Mucoadhesive and Therapeutic Treatments

Buccal delivery using bigels is currently being explored, especially for treating periodontal disease. Periodontal diseases, i.e., chronic afflictions caused by bacteria accumulation in dental plaque, causing inflammation, are widespread [66]. Mucoadhesive hydrogel formulations are useful in this application because of high patient compliance, but are unable to deliver lipophilic drugs [66]. O/H bigels can therefore bridge this gap by providing the patient the benefits of hydrogel applications, while also carrying lipophilic drugs like metronidazole [66]. A porcine buccal mucosa ex vivo model was used to assess the effectiveness of metronidazole bigels composed of aerosil 200, Tween 80, tocopherol acetate, and linseed oil organogels, and sodium alginate, glycerol, and ethyl alcohol hydrogels. The bigels were the most effective local delivery method, compared to separate hydrogels and organogels [66]. In fact, the bigel formulation exhibited a high initial permeability rate for good therapeutic effects, and then moderate retention in the tissue for continued release with less risk for cytotoxicity [66]. Nonsteroidal anti-inflammatory drugs (NSAIDs) are used, in addition to antibiotics, in moderate and severe cases of periodontitis, to ease swelling and inflammation [67]. Ibuprofen was loaded into the oleogel portion of a bigel (Carbopol^®^ and Transcutol^®^ P hydrogel/TegoSoft^®^ CT and Compritol^®^ oleogel). The rheological and bioadhesive properties, and in vitro drug release, were influenced by gel formulations. These parameters could as such be modulated to ensure the delivery of ~95% of the loaded ibuprofen over 6 h compared to its respective individual hydrogel (60%) and oleogel (80%) formulations [67]. Since ibuprofen is lipophilic, the bigel could more effectively deliver the drug, while still providing the optimal mucoadhesive effects of the hydrogel [67].

Mucoadhesion is also a key characteristic for local drug delivery through nasal and vaginal routes. In nasal delivery, hydrogel systems are more easily functionalized than organogels during fabrication to increase biocompatibility, biodegradability, porosity, and mucoadhesion [56]. Thiol groups have been added to polymer chains to increase mucoadhesion, and this strategy can be utilized to develop mucoadhesive bigels [56]. The treatment or prevention of sexually transmitted diseases is effectively carried out through vaginal delivery. Sexual transmission of HIV is the most common transmission route, and topical microbicides have been investigated as a preventative treatment [57]. A bigel for the co-delivery of maraviroc (a specific entry inhibitor) and tenofovir (a nucleotide reverse transcriptase inhibitor) was produced and showed potential for long-term pre-exposure prophylaxis of HIV transmission [57]. This was concluded due to the good anti-HIV activity displayed. At the same time, the bigel did not cause cytotoxicity, or inhibited the normal vaginal microflora, and was compatible with the rectal epithelium [57]. This microbicidal gel relied on drug diffusion, but other release triggers have been explored to increase treatment efficacy [57]. For example, an enzyme-responsive bigel (HPMChydrogel/Span 60 and Tween 80 and soybean oil oleogel) was developed and loaded with maraviroc and tenofovir microsphere carriers built of chitosan crosslinked with sodium tripolyphosphate [68]. The degradation of the carriers by acid phosphatase, an enzyme present in seminal fluid, was measured. The release exhibited first-order kinetics in the presence of the enzyme [68]. This strategy for reducing HIV transmission allows for more drug release during intercourse, increasing the efficacy of the pre-exposure prophylactic treatment [68]. In addition to carrying specific enzymes that can trigger the release of enclosed molecules, seminal fluid also increases vaginal pH. Therefore, pH-sensitive formulations are also considered efficacious in HIV prophylaxis. Bigels formulated using different proportions of polymers (pectin, chitosan, and hypromellose) and loaded with tenofovir were tested as potential pH-sensitive bigels [55]. Pectin-containing bigels proved to be the most effective of the different experimental bigels due to pH-dependent swelling and enhanced mucoadhesion [55]. These bigels provided the fastest release of tenofovir in the presence of seminal fluid [55].

### 3.3. Other Therapeutic Effects

Finally, bigels have been shown to improve the therapeutic effect of drugs due to modification in the release profile. Quercetin has a reported therapeutic effect on non-alcoholic fatty liver disease. However, it also has a marked adverse effect on male fertility [47]. Bigels prepared with cottonseed and cannabis oil, sodium alginate, and ferula gum were optimized and tested on an in vivo mouse infertility model [47]. This bigel formulation showed a therapeutic effect on liver damage caused by non-alcoholic fatty liver disease, with limited negative effects on male fertility (e.g., maintenance of serum testosterone levels, decrease in sperm DNA fragmentation, and increased sperm count, motility, and morphology) [47]. Bigels showed synergistic effects with the therapeutic agent, and therefore resulted in improved treatment [47,53,58,59]. Synergy between the carrier and drug used for treatment should be further investigated to potentially improve other treatment outcomes. Drugs can potentially be incorporated into the carrier formulation (e.g., polymers functionalized with active agents) to further improve drug delivery efficacy (e.g., olive oil to soothe skin in bigels for acne treatment).

## 4. Current Food Applications

Bigels have been widely studied in pharmaceutical applications, as described above. However, in food research, their applications are emerging but still limited. Food-grade bigels are bound by distinct requirements and challenges that differ from those of the pharmaceutical industry. Ingredients must be food-grade, widely available, compliant with regulatory requirements, and ideally cost-effective. Foods are inherently complex systems, which often undergo strenuous processing conditions (e.g., high shear, high temperatures) during production that could potentially impact the bigel’s integrity and properties [9]. Additionally, consumers have strong opinions about the ingredients in their food. Therefore, bigels must be formulated with consumer preferences in mind (e.g., clean-label, plant-based) [73]. Due to these additional requirements, the use of bigels in food is viable, but has not been translated to commercial applications. Research on the food applications of bigels has been mostly focused on two main application categories, i.e., solid fat replacers (SFRs) and delivery systems for probiotics, bioactive molecules, and beneficial free fatty acids. Table 2 summarizes different bigel systems researched for food applications. 

### 4.1. Solid Fat Replacement

Solid fat replacement is a major consumer trend that, until recently, has been primarily addressed with the use of modified carbohydrates or proteins and organogels [16,18]. The growing trend towards healthier food products, e.g., products with less saturated fats, has prompted research into formulating alternative foods with high nutritional quality that also maintain the palatability, texture and functionality of conventional solid fats [9]. Reviews on the different roles fat fulfills in food products demonstrate the multitude of potential interactions of fat in the food matrix and its implications for food structure and overall quality. This complexity highlights the difficulty of replacing solid fats [18,92,93]. Although oleogels (edible organogels) have potential use as SFRs, factors such as component compatibility, low resemblance of textural properties, and limited functionality in high-fat products have limited their applications as such. Bigels can be leveraged to overcome some of these drawbacks due to their biphasic nature [9]. In particular, animal and solid fats used in baked goods are key examples of products where creative SFRs are needed.

Initially, SFR bigels have been produced for general use, with the research focussing primarily on the textural and rheological properties of the bigels to determine their suitability as functional food ingredients. A hot emulsification technique was used to prepare a bigel based on gelatin and glycerol monostearate (GMS), with lecithin and glycerol as a surfactant and co-surfactant, respectively [38]. Multiple formulations were tested, and an optimal relationship was found between the structuring agent concentration and gel hardness and stability, with lecithin addition (up to 5%_wt_) enhancing gel strength and resulting in bicontinuous gels [38]. These preliminary composition-structure–function relationships could be used as guidelines for the further development of additional bigel SFRs. The optimal formulation (2%_wt_ gelatin, 25%_wt_ GMS, and up to 5%_wt_ lecithin) was a suitable SFR with the additional benefit of being less prone to oxidation than saturated fats [38]. Gels with a high (>50%) lipid fraction were also produced from gelatin and rice bran wax in highly oleic sunflower oil [74]. These H/O bigels were highly stable, compared to the separate gelatin hydrogel and rice bran wax oleogel controls, against repeated freeze–thaw cycles [74]. Bigels had 50% less total liquid loss and no rancidity following storage for up to 30 days at 4, 25, and 40 °C [74]. This research provides insights into the potential use of bigels as SFRs in frozen food products.

Animal fats significantly contribute to the saturated lipid content of food products. To address this issue, the manufacture of meat products has been shifting to formulations with reduced fat and improved fatty acid profiles. Bigels have been investigated as SFRs in meat products such as burgers and sausages. In coarsely ground fully cooked sausages, four bigel formulations were tested as pork back fat replacers [75]. The bigels, prepared with different ratios of gelatin/rice bran wax and high oleic soybean oil (at two gelator concentrations for both the hydro and oleo-component), were tested for the complete replacement of pork back fat [75]. The bigels resulted in no changes in the texture profile of the meat products. Furthermore, the results of the sensory evaluation indicate that most of the bigel sausage formulations had acceptable sensory qualities [75]. The bigels exhibited discrete semi-solid particles throughout the sausage, which may also be desirable in other comminuted meat product applications [75]. In fermented sausages, gelatin and κ-carrageenan hydrogel/monoglycerides and olive oil oleogel bigels were designed for partial pork back fat substitution [76,94]. Two bigels (60 and 80% hydrogel phase) were added at 9% of the sausage weight. Bigel substitution did not affect water activity, or the composition of the bacterial populations, during the fermentation and ripening steps [76]. A consumer sensory evaluation of these products did not reveal significant differences in color, texture, juiciness, flavor, taste, or overall acceptability between control and treated sausages [76]. In a third study, bigels were used as SFRs in a model burger formulation. Bigels of starch hydrogel/ethylcellulose and sunflower oil oleogel at different oleogel fractions (25% (O/H), 50% (H/O), and 75% (H/O)) were assessed as SFRs [77]. These bigels showed good thermal stability and mechanical properties at a 75% oleogel fraction in the burger model [77]. Additionally, all the bigel burger formulations showed good cooking characteristics and acceptable sensory properties at up to 50% animal fat replacement [77]. However, in contrast to the bigels used in sausage applications, lipid oxidation was more noticeable when bigels were added to the burger recipes [77]. Incorporating antioxidants could address this challenge for foods that require cooking at high temperatures.

In bakery products, fat plays an important role in structure development while mixing, fermenting and baking. It is also crucial to the mouthfeel and texture of the final product. Cookies, in particular, are high-fat products where solid fat is necessary for proper structure development. When oils are used in these products instead of solid fat, the cookie’s dimensions and hardness are negatively affected. In sugar-snap cookies, the fat content was reduced by replacing commercial shortening with a bigel [78]. Up to 50% of the fat content (100% replacement of shortening) was reduced using a bigel (gelatinized corn starch hydrogel/candelilla wax and canola oil oleogel) [78]. The cookies prepared with the bigel displayed lower hardness and higher firmness than cookies formulated with shortening when tested using a TPA [78]. These differences are considered desirable for cookies, since they result in softer cookies that can be handled while maintaining product integrity [78]. Notably, using gelatinized starch in the bigel resulted in increased in vitro starch digestibility and glycemic index [78]. Alternative hydrogelators were investigated in another cookie formulation to avoid a glycemic index increase. Beeswax and canola oil oleogels were combined with either sodium alginate or carboxymethylcellulose (CMC) hydrogels and used as SFRs [19]. While oleogels alone had a negative impact on texture, bigel fat replacers resulted in a hardness similar to control cookies [19]. Additionally, plastic fats, such as cream, are common bakery ingredients. Chitosan-based hydrogels and glycerol monolaurate and medium chain triglycerides (with cinnamaldehyde as a crosslinker) oleogels were used to prepare O/H bigels that could be used as an alternative to cream [46]. The bigels were pH-sensitive, and at optimal pH conditions (~pH 3.8) were homogenous (no visible phase separation) [46]. The addition of Span 80 also improved the phase distribution and texture of the cream analogues [46].

“Healthier” vegetable oil-based spreads can also be prepared using bigels. Different hydrogel structuring agents (gelatin, agar, and collagen) and the effect of lecithin were explored in the preparation of a bigel-based oil spread enriched with lingonberry pomace [79]. All prepared bigels were stable throughout two-week refrigerated storage, and the incorporation of lingonberry pomace provided a considerable amount of dietary fiber (11 g/100 g of spread) [79]. However, the pomace addition also contributed to a grainy texture, so more research can be conducted to improve the sensory attributes of the spread while maintaining the nutrient profile [79]. Food-grade bigels were also studied as foods that could potentially meet the needs of dysphagia patients [83]. Using a collagen hydrogel/carnauba wax and sunflower/olive pomace oil oleogel produced a bigel that was safe for dysphagia patients. It provided a substantive amount of protein, which may reduce the risk of malnutrition [83]. Palliative care dysphagia patients can safely ingest products with “nectar-like”, “honey-like” and “spoon-thick” consistencies. The prepared bigels fell into the “honey-like” and “spoon-thick” consistency categories based on viscosity measurements [83].

Cocoa butter has a unique triglyceride structure that is highly desirable in confectionary. However, due to its high price and lack of adequate substitutes, research into cocoa butter substitutes is emerging [80]. A bigel was produced with sodium alginate hydrogels and beeswax and grape seed oil oleogels at different ratios [80]. The thermal and chemical properties of several formulations were used to select the ideal O:H ratio (95:5), and this bigel was used to prepare compound chocolate at four replacement levels (0, 15, 30, and 45%) [80]. Up to 15% replacement could be achieved without significant differences compared to the control. This bigel, therefore, is promising as a basis upon which to produce cocoa butter substitutes [80].

The transition towards plant-based foods and food ingredients is another application in which bigels might play an important role. Plant-based bigels could replace animal-derived fats and plant-based structuring agents. Previously researched SFRs contain gelatin as a hydrogelator, and so alternative hydrogelators also need to be assessed. To this end, bigels were produced from sodium alginate and κ-carrageenan, as well as rice bran wax and soybean oil [81]. The O:H ratio, and the addition of monoglycerides, were assessed to aid in developing a plant-based, semi-solid fat replacement [81]. An 8:2 O:H ratio, with 2% monoglyceride, was determined to be a suitable plant-based replacer for semi-solid fats [81]. A tunable oral sensation and controlled gastrointestinal release profile are also in demand. Fat plays a significant role in food mouthfeel, and therefore the assessment of bigel tribology is vital, although vastly under-researched. The structural, rheological, tribological, flavor release, and delivery properties of bigels prepared from konjac glucomannan–gelatin hydrogels and stearic acid oleogels were assessed [82]. The mass ratio of konjac glucomannan to gelatin resulted in a change in structure, rheology, and tribology with increases in konjac glucomannan resulting in an enhanced storage modulus and yield stress, and decreases in the structure-recovery properties of the bigel [82]. At oral temperatures, viscoelastic modulus and viscosity decreased with increasing konjac glucomannan content [82]. The oral sensation of the bigel was therefore modulated by modifying the formulation [82].

Several studies have investigated bigels in 3D printing applications [24,25,84,85,86,95,96]. The phase inversion of the bigel structure is a major focus, as the structure of the bigel (e.g., O/H, H/O, or complex) has been found to drastically affect the printability of bigel inks [24,25,84]. Jiang et al. formulated bigels of gelatin hydrogel/GMS and ethylcellulose oleogels of which the composition was deliberately changed to obtain different bigel types [25]. The O/H bigel had similar hardness and formability to the separate gelatin hydrogel, and possessed ideal rheological properties for decorating purposes (e.g., piping), whereas H/O bigels were more appropriate for 3D printing due to their better thixotropic properties [25]. In HPMC hydrogel/beeswax oleogel bigels, changes in the O:H ratio were also able to produce three distinct structures [24]. The different thermal properties of the hydrogelator and oleogelator extended to the O/H and H/O bigels, generating distinctly different products [24]. H/O bigels exhibited the best printing integrity among the bigel types obtained, while the semi-bicontinuous bigels were the least suitable, possibly due to the inhomogeneity of the system [24]. *Spirulina platensis* protein nanoparticle-based bigels (xanthan gum hydrogel/sunflower wax oleogel) combined two mechanisms (stabilization through a Pickering emulsion and gelation) to improve bigel printing properties [84]. In this system, the selection of the O:H ratios also led to phase inversions in the bigel structure [84]. The incorporation of protein nanoparticles improved bigel printability compared to the control bigel formulation [84]. It was noted that 3D printing significantly decreased the hardness, springiness, cohesiveness, and gumminess of the bigel, and consequently the textural characteristics of the final food product [84]. The thixotropy of the bigels increased with increasing oleogel content, allowing for the deposition of an increasing number of layers to amount to a complete structure [85,86]. Bigels are promising food-grade 3D-printing inks due to their tunability.

### 4.2. Delivery Systems for Labile Compounds

Extensive research has been performed on bigels as drug delivery in pharmaceutical applications, but bigels are also promising delivery vehicles for bioactives in foods. Food-grade bioactive ingredients have specific physiological functions and include polyphenols, polyunsaturated fatty acids, vitamins, and other compounds [97]. Other additives, like probiotics, can also functionalize food and provide additional benefits. However, some bioactive compounds can generate undesirable flavors and textures, which can decrease consumer acceptance and intake [97]. Delivery systems like bigels can enhance these bioactives’ stability, optimize their solubility, improve their biocompatibility, and conceal off-flavors and odors [97]. Especially labile bioactive compounds can benefit from protection against adverse environmental conditions like oxygen, UV light, heat, and extreme pH conditions, all of which are common during food processing, distribution and storage. Therefore, fulfilling consumer demands for functional foods requires effective carriers to deliver significant amounts of bioactives that can withstand challenging conditions throughout the food supply chain [98].

In bioactive delivery, a key concern is the bioaccessibility of the incorporated compounds. In vitro digestive models provide a useful tool for monitoring the availability and uptake of active agents. Bigels developed as SRFs can simultaneously serve a secondary role as bioactive delivery vehicles [82]. Bigel formulation allowed for tailoring the oral sensations of the bigel (gelatin and konjac glucomannan hydrogel/stearic acid oleogel), while also modulating the bioactive release [82]. Hydrogel content was used to control swelling, lipid digestion, and the release of lipophilic actives (i.e., free fatty acids and quercetin) from these O/H bigels [82]. Bigels (κ-carrageenan hydrogel/monoglyceride oleogel) were developed as carriers for ß-carotene, another lipophilic active agent [87]. Consistent with previous research, the O:H ratio determined the bigel structure obtained from O/H to bi-continuous to H/O structures as oleogel content increased [87]. A rising oleogel content positively impacted the UV light stability and in vitro gastrointestinal release of ß-carotene [87]. For bigels (gelatin hydrogel/GMS oleogel) developed for the co-delivery of curcumin and epigallocatechin gallate, oleogelator concentration was varied [60]. The release of curcumin was specifically controlled by the oleogelator concentration, while epigallocatechin gallate release was not [60]. Curcumin was slowly released in the gastric compartment and then rapidly released in the intestine. In contrast, epigallocatechin gallate was quickly released in the gastric juice and then released at a constant rate in the intestine [60]. The co-delivery of active agents, where the release rate can be controlled for each phase, is highly promising for diverse applications in food products. The delivery of lycopene in the gastrointestinal tract via bigels (high acyl gellan gum hydrogel/GMS and beeswax oleogel) has also been assessed. Contrary to other work, where higher oleogel proportions increased the release of ß-carotene, in this study, higher oleogel proportions slowed the release of lycopene [87,88]. These contradictory results for lipophilic bioactives indicate the potential effects of oleogelator and bigel structure on the bioactive release rate.

Bioactive polyunsaturated fatty acids have proven health benefits and are also good candidates for protection and delivery via bigels due to their low oxidative stability [89]. Bigels (CMC hydrogel/monoglyceride oleogel) prepared with three vegetable oils as liquid phases (i.e., coconut, avocado, and pomegranate oil) were investigated for their nutritional attributes [89]. The bigel matrix protected the fatty acids from oxidation [89]. The bigels with avocado oil were then used to enrich yogurt [99]. The presence of bigel-encapsulated avocado oil in the yogurt recipe enhanced the nutritional profile through a significant reduction in the atherogenic and thrombogenic indexes [99]. The free-form avocado oil yogurt had eight-fold less free unsaturated fatty acids than the encapsulated form due to the enhanced protection contributed by the bigel structure [99]. Additionally, the yogurt functionalized with avocado oil bigels modulated obesity-related metabolic pathways (e.g., glycerol release) [99]. In another study, yogurt was functionalized with bigels containing coconut and avocado oils [100]. The yogurt containing bigels was again more nutritionally functional than yogurt containing free-form vegetable oils [100]. Increased adiponectin secretion and reduction in interleukine-6 secretion were observed, further evidence that these yogurts can be used for obesity management [100].

Probiotics must have an optimal environment to maintain their biological viability. Probiotics, to confer a health benefit, should be able to withstand the conditions in the upper part of the gastrointestinal tract to adhere to and colonize the intestinal epithelium [90]. Delivering viable probiotics is challenging since food processing, storage, and eventual ingestion and digestion impact probiotic survival. A whey protein concentrate hydrogel/soy lecithin and stearic acid oleogel bigel was prepared. The bigel and its components’ (particularly the phospholipids) ability to protect probiotics during in vitro digestion was assessed [90]. While the formulated bigel protected the probiotics more effectively than the control formulation (containing no gelators), phospholipids did not affect probiotic survival [90]. Starch and non-starch polysaccharide-based hydrogel bigels containing sunflower oil oleogel were also explored for the delivery of probiotics [101]. This study aimed to co-deliver probiotics and an antibiotic (metronidazole) [101]. Antibiotics can have a negative impact on the gastrointestinal microflora, and the co-delivery of probiotics can mitigate this side effect. The bigels showed diffusion-mediated release of metronidazole, and the bigel-encapsulated probiotics were tolerant to gastric and intestinal conditions [101]. Therefore, this bigel system effectively and simultaneously delivered poorly soluble drugs and probiotics [101].

Food packaging presents another opportunity for bigel-mediated release. Bigel edible coatings can reduce packaging requirements and extend the shelf life of foods [91]. A bigel coating (containing rosemary oil) applied to sardine fillets was able to reduce lipid oxidation, but did not significantly impact microbial growth [91]. The 3D printing of bigels has been used to produce “smart” food packaging materials [96]. A H/O bigel (agar and sweet potato anthocyanin hydrogel/beeswax, glycerol monooleate and sunflower oil oleogel) was extruded onto polyvinylidene fluoride film via 3D printing, and the film was sensitive to spoilage metabolites [96]. The packaging film displayed a color change when volatile amines (e.g., trimethylamine) were produced as spoilage metabolites during the storage of beef and salmon [96]. Bigels can therefore present opportunities for use in novel techniques, such as 3D printing and “smart” food packaging, as a carrier for sensors and shelf life-extending molecules.

## 5. Future Directions and Conclusions

Bigels present numerous benefits compared to other gelled systems like individual hydro- and oleogels. Further research is necessary to fully unlock the potential of these systems, since there are still gaps in knowledge on the mechanisms of bigel formation, their interaction with other food components, and the development of effective scale-up methods [9]. While bigels are more complex than their individual oleogel and hydrogel phases due to the interactions between the phases, information can still be gleaned from the extensive and more complete research efforts made into the separate hydrogels and oleogels. Previous work into these systems—and lessons from the pharmaceutical industry—can be leveraged to further develop more sophisticated bigels for food uses.

A relatively novel approach to bigel preparation is leveraging all components of the systems. In most cases, when considering bigels for treatment (especially when incorporating bioactives or drugs), the components are selected to protect/release the encapsulated material. However, by strategically selecting the organic liquid phase of the bigel, technological and health features can be tailored more effectively. For example, the use of fish, olive, or sesame oil as organogel liquid phases in numerous topical applications provides both structural (as the organogel liquid phase) and functional (e.g., omega-3 free fatty acids) features translatable to food applications [10,26,48,53,54,56,57,60,62,65,76,77]. In beeswax oleogels, ß-carotene was incorporated as an antioxidant, but it also had a significant impact on the oleogel hardness [102]. Unsaturated fatty acid-rich vegetable oils were used to produce bigels for functionalized yogurt [89,99,100]. These examples suggest that bigel component functionality can expand beyond modulating mechanical/rheological properties and can extend to therapeutic functions.

Also, gelator choice can be utilized strategically to gain greater therapeutic effects (or health benefits) or to modify release rates. For example, a method for synthesizing protein–antioxidant complexes has been developed using gelatin, gallic acid, and catechin [103]. These functionalized gelatin complexes could provide both structural and bioactive functions if incorporated into a bigel [103]. Using multiple gelators to co-structure the gels can modulate the mechanical properties of the gels and even provide additional release triggers [98,104]. Co-polymer gels (e.g., gelatin–agar-based co-hydrogels) can be achieved by tailoring the composition of bio-compatible gelators [54]. Co-polymer gels focused on polysaccharide-based co-hydrogels of sodium alginate and κ-carrageenan were incorporated into rice bran wax oleogels to produce plant-based bigels [81]. If the linkages between polymers are sensitive to specific triggers (e.g., enzymes or pH), bigels with co-hydrogels would have more versatile uses. Gelators can also provide a therapeutic effect. For example, a greater effort has recently been made to develop both pre- and probiotic functional food products. Dietary fiber and oligosaccharides have prebiotic effects on gut microbiota and have found use as gelators [105]. Bigels could encapsulate probiotics in a prebiotic gelator matrix, potentially improving their beneficial effects, stability, and viability.

Low-value by-products from existing food processing operations that would otherwise end up either as food waste or low-value feed can also be leveraged to make bigel incorporation more attractive to the food industry. Rice bran wax is a popular oleogelator due to its upcycled status as a processing by-product [29,40,74,81]. By-products such as lingonberry pomace have also been functionalized by utilizing bigels as a carrier to improve the sensory attributes of the pomace [79].

**Figure 5:**
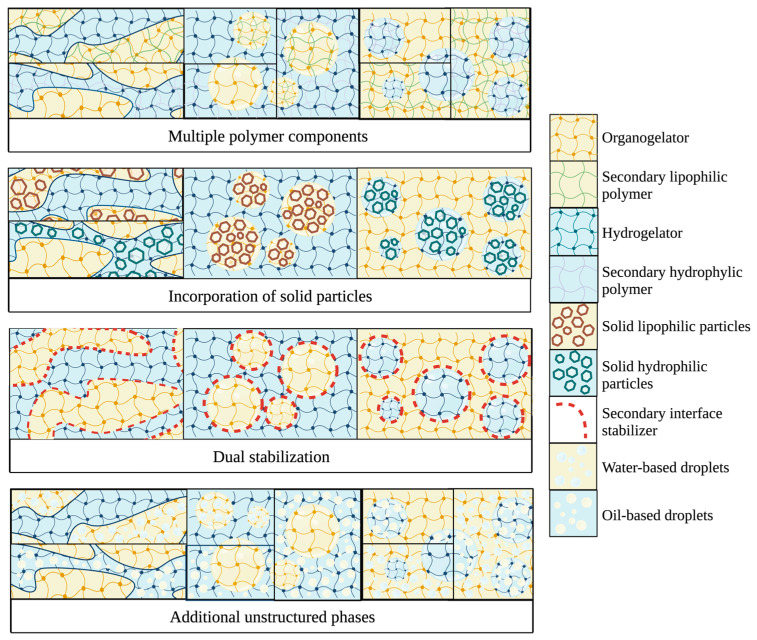
Schematic diagram of potential “unconventional” bigels with added complexity. Illustration created with biorender.com.

Unconventional bigels, with additional colloidal phases and modifications, are under-researched, but present many opportunities for use in foods. Figure 5 illustrates some potential configurations for unconventional and complex bigels. Colloids other than gels could be incorporated into bigels to exploit their unique physicochemical properties [9]. The production of complex bigels is possible by incorporating unstructured water or oil phases into the hydrogel or oleogel phase [9]. For example, a topical cosmetic bigel-based cream was prepared by combining an emulgel (hydrogel and unstructured oil) and a hydrogel, and then combining the emul-hydrogel with an oleogel [52]. In another case, an oleogel emulsion (soy lecithin, stearic acid, and soybean oil oleogel with water) was prepared, and then combined with a hydrogel to form a bigel [106]. These systems result in higher water or lipid contents while still relying on a double-gelled structure for stability. Additional aspects such as using multiple polymers (as discussed above), additional stability conferred by membranes, solid particle addition for increased stability, and combinations of these strategies can be incorporated for increased tunability, stability, and more versatile release.

The intersection of delivering both adequate nutrition and health benefits through food is sometimes referred to as “food as medicine” [107]. Information from the pharmaceutical industry can be applied to foods to produce these functional “food as medicine” products. A main area requiring further development is the use of specific triggers for release. A substantial amount of the currently published bigel drug delivery research relies on diffusion-mediated drug release. While diffusion release is necessary for some treatments, there is a wide range of environments in food. Additionally, food products often do not get consumed immediately, and the degradation of bioactive compounds during storage is an important factor to consider. Thermal triggers can be explored to deliver bioactive compounds during cooking or heating. Antioxidants may be used if exposure to oxidative conditions is expected, like during deep-frying processes, to reduce lipid oxidation in food products. Processes that involve pH changes could also be leveraged, such as pickling or other fermentation methods. Pectin and alginate are pH-sensitive polymers that can degrade at food-relevant pHs [97,108]. Finally, functional polymers could be incorporated into food products to further improve gastrointestinal delivery. For example, mucoadhesive polymers could be used to encapsulate materials to be delivered and retained in the intestinal mucosa (e.g., probiotics).

Research on the effects of bigel components on final properties can also be utilized for the purposeful design of bigels. It was found that the dispersed phase can act as an active filler, which increases bigel strength. At the interface, organogelators interact with water and increase the affinity between the phases. The O:H ratio has the biggest influence on physical properties, but organogelator concentration and mixing rate also have an effect. These known bigel properties, in combination with novel approaches to bigel formulation in foods, can be further investigated to provide highly efficient functional food products for consumers. Bigels have been well studied and applied in pharmaceutical applications, but they have untapped potential in food applications. More research is needed to make cost-effective, functional bigels for application in functional foods.

## Figures and Tables

**Table 1:** Bigel systems (main components) developed for pharmaceutical applications.

Application	Oleogel		Hydrogel	O:H Ratio	Morphology	Incorporated Drug	Ref.
	Oleogelator	Solvent	Hydrogelator				
Fundamental	Stearic acid	Rice bran oil	Tamarind gum	80:20	H/O	Moxifloxacin	[41]
				40:60	O/H		
	Span 60	Sesame oil	Gelatin	12:88	Complex	Ciprofloxacin	[33]
	Candelilla wax	Sesame oil	Guar gum	75:25	H/O	Moxifloxacin	[61]
	Beeswax	Fish oil	Sodium alginate/ Hydroxypropyl Methylcellulose (HPMC)	10:90	H/O	Imiquimod	[53]
				30:70			
				50:50			
Cosmetic	Span 60	Sweet almond oil	Carbopol^®^	10:90	O/H	None	[21]
	Cholesterol	Liquid paraffin		5:95			
	Zinc stearate	Paraffin		7:93			
	Beeswax	Fish oil	Carbopol^®^	50:50	Not reported	Coenzyme Q10	[58]
Antifungal treatment	Tween 80	Castor oil	Sodium alginate	30:70	Not reported	*Bidens tripartita* essential oil	[62]
	Polyethylene	Liquid paraffin	Poloxamer 407	10:90	O/H	Ciclopirox olamine and terbinafine hydrochloride	[63]
				20:80			
				30:70			
				40:60			
Transdermal (skin cancer, antimicrobial)	Span 60	Olive oil	Carbopol^®^	40:60	No bigel	Doxycycline hyclate	[64]
				30:70	Complex		
				20:80			
	Beeswax	Fish oil	Carbopol^®^	10:90	H/O	Imiquimod	[59]
				30:70			
				50:50			
	Beeswax	Olive oil	Hydroxyethylcellulose	40:60	Complex	Povidone- Iodine	[65]
				60:40	H/O		
Periodontitis	Tween 80	Linseed oil	Sodium alginate	30:70	O/H	Metronidazole	[66]
	Compritol^®^	TegoSoft^®^ CT	Carbopol^®^	50:50	O/H	Ibuprofen	[67]
HIV prevention	Tween 80	Palm olein	Hyaluronic acid	50:50	O/H	Tenofovir and Maraviroc	[57]
				40:60			
				60:40			
	Span 60 and Tween 80	Soybean oil	HPMC	50:50–90:10	Not reported	Encapsulated Tenofovir and Maraviroc	[68]
	Span 60	Sesame oil	Pectin, chitosan, or HPMC	80:20	H/O	Tenofovir	[55]
Male Infertility	Stearic acid	Cottonseed & cannabis oils	Sodium alginate and ferula gum	Not reported	H/O	Querecetin	[47]

**Table 2:** Bigel systems developed for food applications.

Application	Oleogel		Hydrogel	O:H Ratio	Morphology	Ref.
	Oleogelator	Solvent	Hydrogelator			
Solid Fat Replacer	Glycerol monostearate (GMS)	Canola oil	Gelatin	50:50	Complex	[38]
	Rice bran wax	High oleic soybean oil	Gelatin	40:60–70:30	O/H	[74]
Solid Fat Replacer (sausage)	Rice bran wax	High oleic soybean oil	Gelatin	70:30	Not reported	[75]
				60:40		
	Monoglycerides	Olive oil	Gelatin and k-carageenan	40:60	O/H	[76]
				20:80		
Solid Fat Replacer (burger)	Ethylcellulose	Sunflower oil	Starch	25:75 50:50–75:25	O/H	[77]
					H/O	
Solid Fat Replacer (cookie)	Candelilla wax	Canola oil	Gelatinized corn starch	50:50	Not reported	[78]
	Beeswax	Canola oil	Sodium alginate or carboxymethylcellulose (CMC)	50:50	H/O	[19]
Solid Fat Replacer (spread)	Carnauba wax	Sunflower and olive pomace oil	Gelatin, agar, or collagen	60:40	Not reported	[79]
Solid Fat Replacer (chocolate)	Beeswax	Grapeseed oil	Sodium alginate	99:1	H/O	[80]
				5:95		
				90:10		
Solid Fat Replacer (plant based analogue)	Rice bran wax	Soybean oil	Sodium alginate and k-carageenan	70:30	H/O	[81]
				80:20		
Solid Fat Replacer (plant based analogue), Bioactive and Flavor Carrier	Stearic acid	Soybean oil	Konjac glucomannan and gelatin	50:50	H/O, complex, and O/H depending on konjac:gelatin mass ratio	[82]
Plastic Fat Replacer	Glycerol monolaurate	Medium chain triglycerides and cinnemaldehyde oil	Chitosan	20:80–40:60	O/H	[46]
Dysphagia Product	Carnauba wax	Sunflower and olive pomace oil	Collagen	40:60	O/H	[83]
				50:50		
				60:40	H/O	
3D Printing Ink	GMS and Ethylcellulose	Soybean oil	Gelatin	20:80	O/H	[25]
				40:60		
				42:58	Complex	
				44:56		
				46:54–80:20	H/O	
	Beeswax	Soybean oil	Hydroxypropyl methylcellulose (HPMC)	20:80–50:50	O/H	[24]
				55:45	Complex	
				60:40	H/O	
				80:20		
	Sunflower wax	Soybean oil	Xanthan gum	20:80–54:46	O/H	[84]
				56:44	Complex	
				58:42		
				60:40	H/O	
				80:20		
	Beeswax and GMS	Soybean oil	Gellan gum	30:70–60:40	O/H	[85,86]
				65:35	Complex	
				70:30	H/O	
				80:20		
Bioactive Carrier	GMS	Corn oil	k-carageenan	25:75–50:50	O/H	[87]
				60:40	Complex	
				75:25	H/O	
	GMS	Corn oil	Gelatin	50:50	O/H	[60]
	GMS	Soybean oil	High acyl gellan gum	10:90–60:40	O/H	[87,88]
	Geleol	Coconut, avocado, or pomegranate oil	CMC	Not reported	Not reported	[89]
	Soy lecithin and stearic acid	Soybean oil	Whey protein concentrate (80)	80:20	H/O	[90]
	Span 40	Sunflower oil	CMC, sodium alginate, or maltodextrin	50:50	O/H	[91]
Active Packaging	Monoglycerides	Sunflower oil	Gelatin	20:80	O/H	[92]
	Beeswax and glycerol monooleate	Sunflower oil	Agar	80:20	H/O	[93]

## Data Availability

Data sharing is not applicable.
